# Drug Overdose Deaths Among Women Aged 30–64 Years — United States, 1999–2017

**DOI:** 10.15585/mmwr.mm6801a1

**Published:** 2019-01-11

**Authors:** Jacob P. VanHouten, Rose A. Rudd, Michael F. Ballesteros, Karin A. Mack

**Affiliations:** ^1^Division of Analysis Research and Practice Integration, National Center for Injury Prevention and Control, CDC; ^2^Division of Unintentional Injury Prevention, National Center for Injury Prevention and Control, CDC.

The drug epidemic in the United States continues to evolve. The drug overdose death rate has rapidly increased among women (*1*,*2*), although within this demographic group, the increase in overdose death risk is not uniform. From 1999 to 2010, the largest percentage changes in the rates of overall drug overdose deaths were among women in the age groups 45–54 years and 55–64 years (*1*); however, this finding does not take into account trends in specific drugs or consider changes in age group distributions in drug-specific overdose death rates. To target prevention strategies to address the epidemic among women in these age groups, CDC examined overdose death rates among women aged 30–64 years during 1999–2017, overall and by drug subcategories (antidepressants, benzodiazepines, cocaine, heroin, prescription opioids, and synthetic opioids, excluding methadone). Age distribution changes in drug-specific overdose death rates were calculated. Among women aged 30–64 years, the unadjusted drug overdose death rate increased 260%, from 6.7 deaths per 100,000 population (4,314 total drug overdose deaths) in 1999 to 24.3 (18,110) in 2017. The number and rate of deaths involving antidepressants, benzodiazepines, cocaine, heroin, and synthetic opioids each increased during this period. Prescription opioid–related deaths increased between 1999 and 2017 among women aged 30–64 years, with the largest increases among those aged 55–64 years. Interventions to address the rise in drug overdose deaths include implementing the CDC *Guideline for Prescribing Opioids for Chronic Pain* (*3*), reviewing records of controlled substance prescribing (e.g., prescription drug monitoring programs, health insurance programs), and developing capacity of drug use disorder treatments and linkage to care, especially for middle-aged women with drug use disorders.

Mortality data for U.S. residents were obtained from the 1999–2017 National Vital Statistics System,* which is based on information from all death certificates filed in the 50 states and the District of Columbia. Deaths of nonresidents (e.g., nonresident aliens, nationals living abroad) were excluded. Mortality data were provided to CDC’s National Center for Health Statistics through the Vital Statistics Cooperative Program and coded according to the *International Classification of Diseases, Tenth Revision* (ICD-10). Analyses were restricted to deaths with an underlying cause of death based on the following ICD-10 codes for drug overdoses: X40–X44 (unintentional), X60–X64 (suicide), X85 (homicide), and Y10–Y14 (undetermined intent). Among deaths with drug overdose as the underlying cause, the type of drug involved was based on ICD-10 codes for antidepressants (T43.0–T43.2), benzodiazepines (T42.4), cocaine (T40.5), and opioids (all T40.0–T40.4 and T40.6, including those for heroin [T40.1], prescription opioids [T40.2–40.3], and synthetic opioids, excluding methadone [T40.4]). Deaths involving more than one type of drug were counted in multiple categories. Crude rates are reported as deaths per 100,000 population. Percent change was calculated on unrounded rates. Joinpoint regression^†^ was used to test the significance of overdose trends from 1999 to 2017. Annual percentage change estimates that were statistically significant (p<0.05) are presented to indicate the magnitude and direction of significant trends. Age distribution changes in drug-specific overdose deaths were calculated by 5-year age groupings, with average age of death analyzed for drug type for the years 1999 and 2017.

Among women aged 30–64 years, the crude drug overdose death rate increased 260%, from 6.7 deaths per 100,000 population (4,314 total drug overdose deaths) in 1999 to 24.3 (18,110) in 2017 (Figure 1). The rate of drug overdose deaths involving any opioid increased 492%, from 2.6 per 100,000 population in 1999 to 15.5 in 2017 (data not shown). During this time, rates of drug overdose deaths increased for those involving synthetic opioids (1,643%), heroin (915%), benzodiazepines (830%), prescription opioids (485%), cocaine (280%), and antidepressants (176%). Significant inflection points in trends of crude death rates of drug overdoses by drug indicate an increasing annual percentage change for all drugs except cocaine, for which crude death rates significantly decreased from 2006 to 2009.

**FIGURE 1:**
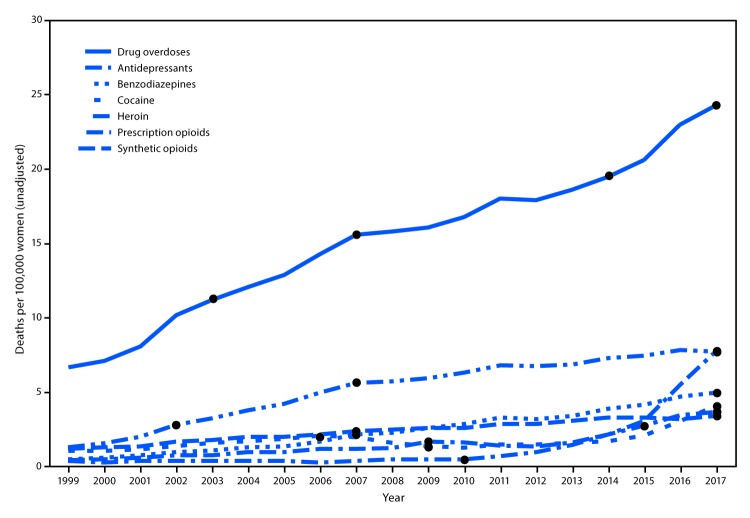
Drug overdose deaths* (unadjusted) per 100,000 women aged 30–64 years, by involved drug or drug class — National Vital Statistics System (NVSS), 1999–2017^†,§^ * Drug overdose deaths were identified using International *Classification of Diseases, Tenth Revision* underlying cause-of-death codes X40–X44, X60–X64, X85, and Y10–Y14. The multiple cause-of-death code or codes for each drug were *heroin:* T40.1; *prescription opioids:* T40.2 for natural and semisynthetic opioids (e.g., oxycodone and hydrocodone) and T40.3 for methadone; *synthetic opioids, excluding methadone* (e.g., fentanyl and tramadol): T40.4; *cocaine:* T40.5; *benzodiazepines:* T42.4; and *antidepressants:* T43.0–43.2. Deaths might involve more than one drug; thus categories are not exclusive. ^†^ NVSS mortality data. ^§^ Significant annual percent change indicated by dots. Antidepressants: 1999–2007 = 8.82; 2007–2017 = 3.63; benzodiazepines: 1999–2007 = 18.94; 2007–2017 = 8.91; cocaine: 1999–2006 = 11.59; 2006–2009 = -14.95; 2014–2017 = 36.71; drug overdoses: 1999–2003 = 14.68; 2003–2007 = 8.28; 2007–2014 = 3.31; 2014–2017 = 8.16; heroin: 1999–2010 = 4.17; 2010–2015 = 42.16; 2015–2017 = 12.79; prescription opioids: 1999–2002 = 30.97; 2002–2007 = 15.03; 2007–2017 = 3.47; synthetic opioids: 1999–2009 = 12.64; 2013–2017 = 52.81.

From 1999 to 2017, drug overdose death rates increased by approximately 200% among women aged 35–39 and 45–49 years, 350% among those aged 30–34 and 50–54 years, and nearly 500% among those aged 55–64 years (Figure 2). During 1999, overdose death rates were highest among women aged 40–44 years (9.6 deaths per 100,000 population), whereas during 2017, rates were highest among women aged 50–54 years (28.2).

**FIGURE 2:**
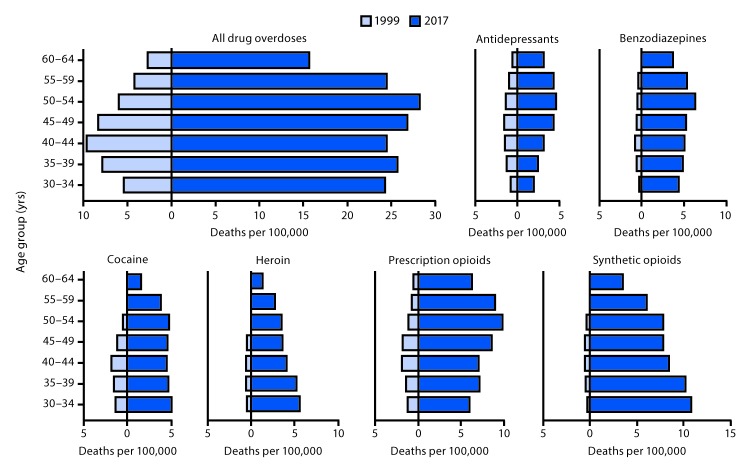
Drug overdose deaths (unadjusted) per 100,000 women aged 30–64 years, by age group and involved drug or drug class — National Vital Statistics System (NVSS), 1999* and 2017^†,§^ * Rates in 1999 for certain age groups are not displayed because counts were <20 deaths. ^†^ NVSS mortality data. ^§^ Drug overdose deaths were identified using *International Classification of Diseases, Tenth Revision* underlying cause-of-death codes X40–X44, X60–X64, X85, and Y10–Y14. The multiple cause-of-death code or codes for each drug were *heroin:* T40.1; *prescription opioids:* T40.2 for natural and semisynthetic opioids (e.g., oxycodone and hydrocodone) and T40.3 for methadone; *synthetic opioids, excluding methadone* (e.g., fentanyl and tramadol): T40.4; *cocaine:* T40.5; *benzodiazepines:* T42.4; and *antidepressants:* T43.0–43.2. Deaths might involve more than one drug; thus categories are not exclusive.

The crude rate of overdose deaths involving antidepressants doubled from 1999 to 2017 among women aged 30–34 years and 40–49 years and increased approximately 300% among those aged 55–59 years, and nearly 400% among those aged 60–64 years. In 2017, rates were lowest among women aged 30–34 years (2.0) and highest among women aged 50–54 years (4.6). Rates of overdose deaths involving benzodiazepines increased in every age group examined (30–34 years, 1,225%; 40–44 years, 534%), with similar rates in 2017 among the 5-year age categories of those aged 35–49 years (range = 4.9–5.3). Similarly, the rate of overdose deaths involving cocaine in 2017 varied little by age category among women aged 30–54 years (range = 4.5–5.0). The crude rate of heroin-related overdose deaths among women aged 30–49 years ranged from 0.4 to 0.6 per 100,000 in 1999; in 2017, rates ranged from 1.3 among women aged 60–64 years to 5.6 among those aged 30–34 years. The crude rate for deaths involving prescription opioids increased from 1999 to 2017 for every age group, with the largest increases (>1,000%) among women aged 55–64 years. The crude rate also increased for every age group for deaths involving synthetic opioids excluding methadone, with the largest increase among women aged 30–34 years (3,500%).

The average age at death from overall drug overdoses among women aged 30–64 years increased by 2.8 years, from 43.5 years in 1999 to 46.3 years in 2017 (Table). The largest increase in average age of death was among cocaine-related deaths (4.7 years), followed by prescription opioid–related deaths (4.5 years). The average age of death among synthetic opioid–related deaths did not change.

**TABLE:** Average age at death among women aged 30–64 years who died of a drug overdose,* by involved drug or drug class — National Vital Statistics System (NVSS), 1999 and 2017^†^

Drug/Drug class involved	Average age at death (yrs)		
	1999	2017	Increase 1999 to 2017
**All drug overdoses**	**43.5**	**46.3**	**2.8**
Antidepressant	44.8	48.9	4.1
Benzodiazepine	44.1	47.1	3.0
Cocaine	40.4	45.1	4.7
Heroin	40.8	43.5	2.7
Prescription opioid	43.3	47.8	4.5
Synthetic opioid	44.2	44.2	0.0

* Drug overdose deaths were identified using *International Classification of Diseases, Tenth Revision* underlying cause-of-death codes X40–X44, X60–X64, X85, and Y10–Y14. The multiple cause-of-death code or codes for each drug were *heroin:* T40.1; *prescription opioids:* T40.2 for natural and semisynthetic opioids (e.g., oxycodone and hydrocodone) and T40.3 for methadone; *synthetic opioids, excluding methadone* (e.g., fentanyl and tramadol): T40.4; *cocaine:* T40.5; *benzodiazepines:* T42.4; and *antidepressants:* T43.0–43.2. Deaths might involve more than one drug; thus categories are not exclusive.

^†^ NVSS mortality data.

## Discussion

From 1999 to 2017, the crude rate of drug overdose deaths among women aged 30–64 years in the United States increased by 260%. The rates of overdose deaths increased for all drug categories examined, with a notable increase in rates of deaths involving synthetic opioids (1,643%), heroin (915%), and benzodiazepines (830%). These findings are consistent with recent reports highlighting an overall increasing trend in deaths involving drugs, especially with shifts in the type of drugs involved (e.g., heroin) (*4*).

Other reports have highlighted the overall increase in overdose deaths and emergency department visits related to drug use, especially among women aged 45–64 years (*1*). In addition to demonstrating the varying drug overdose rate increases by age group, this study determined that the age distribution of decedents shifted from 1999 to 2017, and the average age of women aged 30–64 years dying from drug overdoses increased for every drug class analyzed except synthetic opioids. Prevention programs might need to shift response options as the overdose epidemic experiences demographic shifts. Further, as women progress through life, individual experiences can change in the type of substance used or misused and in the experiences of pain that might result in an opioid prescription (*5*–*8*).

The findings in this report are subject to at least three limitations. First, rate estimates of specific drugs involved with deaths might be affected by factors related to death investigation, such as the substances tested for or the circumstances under which tests are performed. For example, toxicology testing cannot distinguish between pharmaceutical fentanyl and illicitly manufactured fentanyl. Second, drug categories presented are not mutually exclusive, and deaths might have involved more than one substance. Increases in deaths involving certain drugs might be the result of increases in certain drug combinations. Finally, the percentage of deaths with specific drugs identified on the death certificate varies over time. Changes in testing and reporting of drugs might have led to observed increases in some drug entities involved in drug overdose deaths.

Substantial work has focused on informing women of childbearing age about the risk and benefit of the use of certain drugs, particularly for the risk posed by neonatal abstinence syndrome as a result of opioid use during pregnancy (*9*,*10*). The current analysis demonstrates the remaining need to consider middle-aged women who remain vulnerable to death by drug overdose. A multifaceted approach involving the full spectrum of care services is likely necessary. For example, health care providers who treat women for pain, depression, or anxiety can discuss treatment options that consider the unique biopsychosocial needs of women (*2*). Providers can consider implementing the CDC *Guideline for Prescribing Opioids for Chronic Pain* (*3*), and Medicaid programs can also examine whether prescribing of controlled substances to their clients meets established guidelines. Access to gender-responsive substance use disorder treatment services, especially for pregnant women and women with drug use disorders, can reduce harmful outcomes. Overdose deaths continue to be unacceptably high, and targeted efforts are needed to reduce the number of deaths in this evolving epidemic among middle-aged women.

**Summary**

**What is already known about this topic?**

The U.S. drug epidemic is evolving, including among women. Studies have highlighted rising rates of drug overdose deaths among women aged 45–64 years.

**What is added by this report?**

From 1999 to 2017, the death rate from drug overdose among women aged 30–64 years increased by 260%. Drug overdose deaths involving antidepressants, benzodiazepines, cocaine, heroin, prescription opioids, and synthetic opioids all increased. Among women aged 30–64 years, the average age at death for drug overdose deaths increased by nearly 3 years.

**What are the implications for public health practice?**

Overdose deaths continue to be unacceptably high, and targeted efforts are needed to reduce the number of deaths in this evolving epidemic, including those among middle-aged women.

## Footnotes

* https://www.cdc.gov/nchs/nvss/deaths.htm.

† https://surveillance.cancer.gov/joinpoint/.
